# Solid-State Transformations of Mayenite and Core-Shell Structures of C12A7@C Type at High Pressure, High Temperature Conditions

**DOI:** 10.3390/ma16052083

**Published:** 2023-03-03

**Authors:** Sergey A. Gromilov, Anatoly I. Chepurov, Alexander M. Volodin, Aleksey A. Vedyagin

**Affiliations:** 1 Nikolaev Institute of Inorganic Chemistry, Siberian Branch of the Russian Academy of Sciences, Pr. Lavrentieva 3, 630090 Novosibirsk, Russia; 2 V.S. Sobolev Institute of Geology and Mineralogy, Siberian Branch of the Russian Academy of Sciences, Pr. Akademika Koptyuga 3, 630090 Novosibirsk, Russia; 3 Boreskov Institute of Catalysis, Siberian Branch of the Russian Academy of Sciences, Pr. Lavrentieva 5, 630090 Novosibirsk, Russia

**Keywords:** mayenite, carbon coating, core-shell, HPHT, graphite, MgO

## Abstract

Calcium aluminate of a mayenite structure, 12CaO∙7Al_2_O_3_ (C12A7), is widely applicable in many fields of modern science and technology. Therefore, its behavior under various experimental conditions is of special interest. The present research aimed to estimate the possible impact of the carbon shell in core-shell materials of C12A7@C type on the proceeding of solid-state reactions of mayenite with graphite and magnesium oxide under High Pressure, High Temperature (HPHT) conditions. The phase composition of the solid-state products formed at a pressure of 4 GPa and temperature of 1450 °C was studied. As is found, the interaction of mayenite with graphite under such conditions is accompanied by the formation of an aluminum-rich phase of the CaO∙6Al_2_O_3_ composition, while in the case of core-shell structure (C12A7@C), the same interaction does not lead to the formation of such a single phase. For this system, a number of hardly identified calcium aluminate phases along with the carbide-like phrases have appeared. The main product of the interaction of mayenite and C12A7@C with MgO under HPHT conditions is the spinel phase Al_2_MgO_4_. This indicates that, in the case of the C12A7@C structure, the carbon shell is not able to prevent the interaction of the oxide mayenite core with magnesium oxide located outside the carbon shell. Nevertheless, the other solid-state products accompanying the spinel formation are significantly different for the cases of pure C12A7 and C12A7@C core-shell structure. The obtained results clearly illustrate that the HPHT conditions used in these experiments lead to the complete destruction of the mayenite structure and the formation of new phases, which compositions differ noticeably depending on the precursor used—pure mayenite or C12A7@C core-shell structure.

## 1. Introduction

Calcium aluminate with a mayenite structure 12CaO∙7Al_2_O_3_ (usually denoted as C12A7) is the most interesting representative of calcium aluminates and one of the most frequently studied inorganic materials during the last decades. Mayenite as a mineral is known for a long time, and its physicochemical properties and structural characteristics are explored in detail [1,2,3,4,5,6,7]. It was discovered that its cell consists of a cationic framework [Ca_24_Al_28_O_64_]^4+^ and an anionic sublattice, which can contain various anions. In general, the composition of the mayenite phase can be described as 1 unit cell = [Ca_24_Al_28_O_64_]^4+^∙4X^−^, where 4X^−^ can be four singly charged anions (OH^−^, Cl^−^, F^−^), two doubly charged anions (O^2−^), or their various combinations. For a long time, until the beginning of 2000th, the main application area of mayenite was quite traditional for such kind of materials—it was applied in the building industry as a component of cement [8,9]. Later on, mayenite attracted the attention of researchers and manufacturers due to its unique optical properties, which can be significantly enhanced via doping of C12A7 with other cations or substitution of Ca^2+^ in mayenite lattice with such cations [10,11,12,13]. Deposition of an active component on the mayenite surface allows for obtaining efficient catalysts for a variety of catalytic reactions [14,15,16,17,18,19,20,21,22,23,24].

The unique chemical and electrophysical properties of materials with the mayenite structure were discovered about 20 years ago and were studied in detail in numerous works by Prof. Hosono’s group [25,26,27]. In these works, it was demonstrated for the first time that, besides the anions mentioned above, radical anions O^−^ and O_2_^−^ [7,28,29,30,31], hydride ions H^−^ [32,33,34,35], and even simply electrons e^–^ [25,26,27,36,37,38,39] can enter into the composition of mayenite as anions X^−^. In the last case, the obtained material is called eletride and is characterized by metallic conductivity and relatively low work function [40,41,42,43]. Taking into account that the cationic framework of mayenite is very stable, the electride state in an inert medium or a vacuum can exist in a noticeably wide temperature range [38]. It should be noted as well that until now, mayenite is the only known inorganic electride remaining with its properties under such drastic conditions.

The formation of the electride state in mayenite is stipulated by the elimination of sublattice oxygen taking place at high temperatures, which are close to or exceeding the melting temperature for this material. Thereby, such an electride cannot be obtained in a dispersed state via conventional techniques because at elevated temperatures its particles undergo enlargement, agglomeration, and sintering. As we have reported previously [44,45,46,47], the nanoscale size of the mayenite’s oxide core nanoparticles can be maintained via the synthesis of C12A7:e^−^ electride within the core-shell structures of the C12A7@C type. In the C12A7@C structures, the carbon shell serves as a nanoreactor. Its presence in such systems prevents direct contact between the particles of the oxide core and allows for keeping the initial size of the mayenite nanoparticles up to the temperature of 1450 °C, which exceeds its melting temperature. Moreover, the presence of a carbon shell facilitates the significant decrease in the temperature required for the formation of an electride state due to the processes of carbothermal reduction taking place at such temperatures [46,47].

The structural stabilities of various phases of calcium aluminates under High Pressure, High Temperature (HPHT) conditions were investigated in many experimental and theoretical works [48,49,50,51,52,53,54,55,56]. An interest in such studies is connected, first, with the estimation of the possibility to apply these materials under extreme conditions and, second, with a search for a possibility to control their electron, optical and structural properties by the application of the external pressure. At the same time, the information reported in the literature regarding the behavior of mayenite under HPHT conditions is not numerous [48,49,50]. For instance, Zhang et al. demonstrated the effect of irreversible amorphization of the mayenite framework at room temperature and pressure of ~13 GPa [50]. Theoretical estimations of the effects of the nature of X^−^ anion within a such framework, which are presented in the same paper, testify that for the electride state (4X^−^ = 4e^−^), the mayenite framework can be significantly more stable towards the external pressure, and its amorphization will be observed at pressures of ~48 GPa only. According to Murata et al. [49], the use of pressures in a range of up to ~6 GPa in conjunction with high temperatures (up to 1500 °C) is not accompanied by the amorphization of mayenite. Under these conditions, the destruction of the mayenite structure and the formation of more stable calcium aluminates 2CaO∙Al_2_O_3_ (C2A) and 4CaO∙3Al_2_O_3_ (C4A3) are observed. The only paper dedicated to the study of solid-phase reactions involving C12A7 under HPHT conditions with the aim to produce new material was published by Miyakawa et al. [48]. In this paper, the possibility to synthesize solid solutions of the C12A7_(x)_∙S12A7_(1−x)_ compositions, where S12A7 is 12SrO∙7Al_2_O_3_ of mayenite structure, is demonstrated.

The present paper is focused on the study of the behavior of the initial mayenite C12A7 and the core-shell structure C12A7@C under HPHT conditions. The use of graphite and MgO as buffer layers in these experiments allowed for obtaining new information regarding the possible solid-state reaction of mayenite with these materials in the dispersed state. It is worth noting that the exploration of the character of the mayenite interaction with MgO and graphite at high-temperature conditions is of great importance in terms of the possible application of such materials as a supporting substrate to form the electride state from the supported mayenite. The HPHT experiments were performed at a pressure of 4 GPa and a temperature of 1450 °C. The choice of temperature for the HPHT experiments was due to the fact that at conventional experimental conditions (vacuum or argon atmosphere), the structure of mayenite being in a contact with carbon is stable enough at such a temperature [27,46]. Note that the pressure of 4 GPa used in the current experiments was chosen in order to give the possibility for comparison of the obtained results with the data recently reported for mayenite prepared at similar experimental conditions [49].

## 2. Materials and Methods

### 2.1. Synthesis of the C12A7 and C12A7@C Samples

The mayenite samples were synthesized as described elsewhere [45,46,47,57]. Aluminum hydroxide (pseudo-boehmite, Pural SB-1, Condea Chemie GmbH, Hamburg, Germany) and calcium carbonate (special purity, Reachim, Moscow, Russia) served as raw materials. First, calcium oxide was obtained via the calcination of CaCO_3_ in a muffle in the air at 950 °C for 6 h. Then, CaO was gently added to the suspension of aluminum hydroxide in distilled water at room temperature under continuous stirring. The final ratio of the components was equal to the mayenite stoichiometry (12CaO∙7Al_2_O_3_). The mixture stirred for 10 h was filtered, dried at 110 °C, and calcined in a muffle in the air at 600 °C for 6 h. Thus obtained sample was denoted as C12A7-600.

The core-shell sample labeled as C12A7@C-1400 was prepared using the C12A7-600 sample as a precursor. The preparation procedures were as described previously [45,46,47]. The C12A7-600 sample was mixed with polyvinyl alcohol (98%, Reachim, Moscow, Russia) in a ratio of 7:3 and calcined in an argon flow at 1400 °C for 6 h. As was recently reported, the C12A7@C-1400 sample possesses a specific surface area of ~10 m^2^/g and is characterized by a relatively high concentration of localized electrons detectable by the electron paramagnetic resonance technique [46].

### 2.2. HPHT Experiments

**Figure 1:**
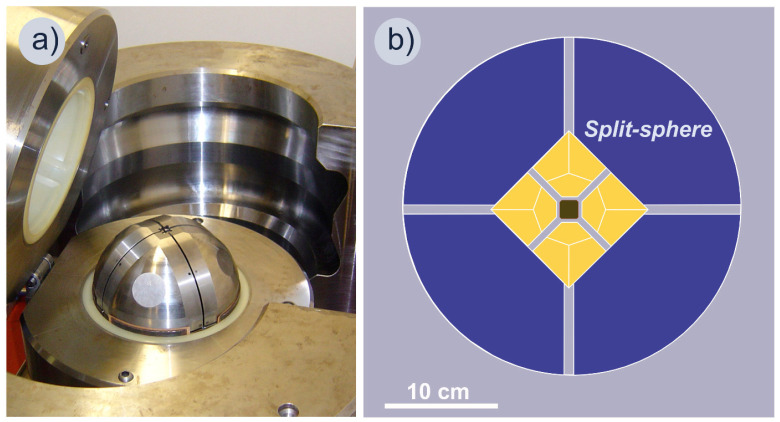
Multiple-anvil high-pressure apparatus of the “split-sphere” type: (**a**) general view; (**b**) schematic vertical section of the power units and the internal stage of the apparatus with a high-pressure cell.

The HPHT experiments were carried out using a multiple-anvil high-pressure apparatus of the “split-sphere” type developed at the Institute of geology and mineralogy, Siberian Branch of the Russian Academy of Sciences (Novosibirsk, Russia) with accordance to the state assignment. Such types of machines are well-known in the literature [58,59,60]. The apparatus does not have a press, and its body consists of two opening semi-blocks, which are enfolded by two flange-type semi-cases (Figure 1a). When closed, a spherical chamber is formed within the semi-blocks which is the space for a multi-anvil spherical guideblock. Two elastic membranes installed inside the semi-blocks separate the guideblock from the apparatus body. Both semi-blocks have channels for pumping oil under the membranes. The loading pressure is transmitted through the membranes to the guideblock. The multi-anvil spherical guideblock named “8/6” consists of the first outer (8) and second inner (6) stages (Figure 1b). The outer stage is a sphere with a diameter of 300 mm consisting of eight separate segments—steel anvils. The top of each anvil is truncated in the form of an equilateral triangle. Compressible plastic gaskets are installed between all anvils of the stage. As assembled, the split-sphere has an octahedral-shaped chamber in its center designed to install six tungsten carbide (WC) anvils. Their truncated tops, in turn, form a parallelepiped-shaped chamber within that is the space for a high-pressure cell (HPC). Pressure in the cell increases as a result of the multiplication of load applied to the spherical outer block and is proportional to the surface ratio between the outer block and the truncated tops of the WC anvils.

The high-pressure cell had a parallelepiped shape with truncated edges, 23.0 × 20.5 mm in size, and was composed of compressed powder refractory oxides ZrO_2_ and CaO. The assembly included a tube graphite heater (0.5 mm thick walls, 10.0 mm inner diameter) placed in the cell center, with graphite and molybdenum discs on the top and at the base used as electrodes. Temperature and pressure were increased at rates of ~200 °C/min and 0.1–0.2 GPa/min, respectively. The temperature was monitored using a PtRh_6_/PtRh_30_ thermocouple. The pressure was estimated using its empirical dependence on oil pressure in the hydraulic system and calibrated by recording changes in the resistance of PbSe, Ba, and Bi. The pressure measurements were accurate to ±0.2 GPa.

**Figure 2:**
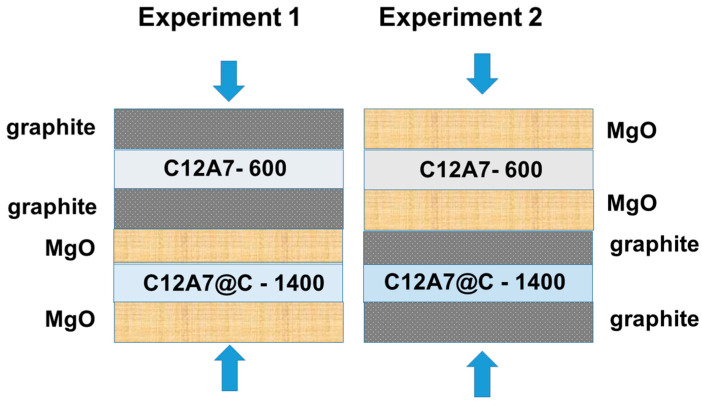
The arrangement of the mayenite samples and buffer layers in the HPHT experiments.

MgO (analytically pure, Krasnyi Khimik, Saint Petersburg, Russia) and graphite (Rizhao Hengqiao Carbon Co., Ltd., Rizhao, China) were chosen as materials for the buffer layers since they are inert enough with regard to mayenite at atmospheric pressure and their contact is not accompanied by the destruction of the mayenite structure up to the temperatures of 1500–1600 °C [27,46]. The disposition of the layers in the experiments is shown in Figure 2. In the first experiment, the C12A7-600 sample was placed between the graphite layers, while the C12A7@C-1400 sample was located between the MgO layers. In the second experiment, the arrangement of the samples and the buffer layers was opposite: C12A7-600 between the MgO layers and C12A7@C-1400 between the graphite layers. The thickness of the graphite layers in both experiments was 1 mm (90 mg). The MgO layers were 2 mm in thickness which corresponds to a weight of 280 mg. The weight of the studied mayenite samples in each layer was 40 mg. The HPHT experiments were performed at a pressure of 4 GPa and a temperature of 1450 °C. The duration of each experiment was 1 h.

### 2.3. Phase Characterization of the Materials

The X-ray characterization of the initial samples (C12A7-600 and C12A7@C-1400) was carried out using a Shimadzu-7000 powder X-ray diffractometer (Shimadzu Corp., Kyoto, Japan) operating with monochromatic CuK_α_ radiation and Ni-filter (Bragg–Brentano geometry). The samples after the HPHT experiments were studied using a Bruker DUO single crystal X-ray diffractometer (Bruker Corp., Karlsruhe, Germany) working with MoK_α_ radiation, graphite monochromator, and charge-coupled device (CCD) detector with a resolution of 512 × 512, D = 60 mm, and 2θ_D_ = 30° (Debye-Scherrer scheme). The used approach includes a recording of a series of Debye-Scherrer patterns (37 pcs.) at different locations of the sample regarding the primary beam and their subsequent summation, as described elsewhere [61,62,63]. During the recording procedure, the sample made a complete revolution around the φ axis in 1 min. The introduction of corrections to the external standard (Si–SRM640) and the transition to the standard type *I*(2θ) was carried out using the DIOPTAS software [64]. X-ray phase analysis and full-profile refinement of the patterns were carried out using the diffraction databases PDF [65] and ICDD [66]. In parallel, the Laue diffraction patterns from the stationary samples were obtained.

## 3. Results and Discussion

### 3.1. Characterization of the Initial C12A7-600 and C12A7@C-1400 Samples

**Figure 3:**
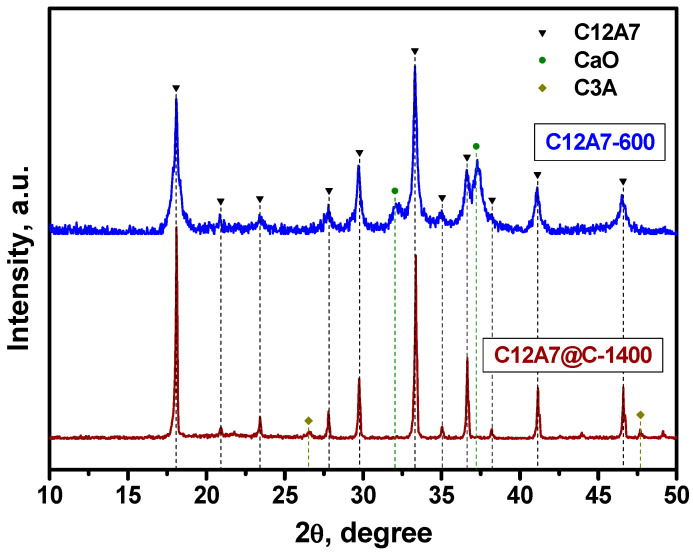
X-ray patterns of the initial C12A7-600 and C12A7@C-1400 samples. The labeled reflections correspond to the mayenite phase, the CaO phase (ICDD card #37-1497), and the C3A phase (ICDD card #38-1429).

First, the two as-prepared samples, C12A7-600 and C12A7@C-1400, were characterized by X-ray diffraction analysis. As follows from Figure 3, the patterns for these samples are noticeably different in the width of the diffraction reflections. This is due to differences in the size of mayenite nanoparticles in such samples and is well consistent with the previously reported data [45,57]. The C12A7-600 sample contains traces of unreacted CaO at this temperature (ICDD card #37-1497), which are absent in the C12A7@C-1400 sample treated at a higher temperature. The impurity C3A phase (ICDD #38-1429) was found in the latter. Refinement of the unit cubic cell parameter showed that in the case of the C12A7-600 sample calcined in an oxygen-containing medium (air), the lattice parameter *a* (12.023 Å) is slightly higher than for the C12A7@C-1400 sample (12.007 Å). The increased value of the lattice parameter at 600 °C can be associated with a high proportion of the C12A7:OH^−^ states in the structure. Similar behavior of parameter a after treatment of mayenite samples in an oxygen-containing atmosphere and in a vacuum was already observed and discussed in the literature [57,67,68]. The reasons for such differences are stipulated by the presence of oxygen-containing anions (X^−^ = OH^−^, O^2−^, O^−^, and O_2_^−^) in the anion sublattice of the C12A7-600 sample and their absence in the case of the electride-state sample C12A7@C-1400 (X^−^ = e^−^), parameter a for which takes a value close to that observed for the electride C12A7:e^−^ [57,69,70].

### 3.2. Characterization of the C12A7-600 and C12A7@C-1400 Samples after the HPHT Experiment between Graphene Layers

**Figure 4:**
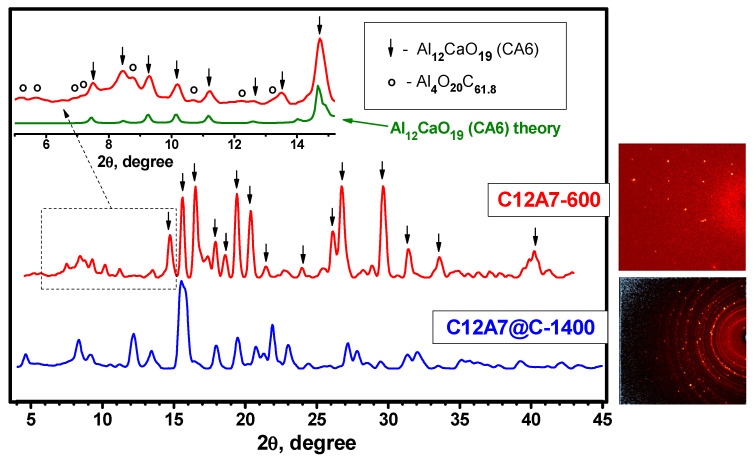
X-ray patterns of the C12A7-600 and C12A7@C-1400 samples after the HPHT experiments with the graphite buffer layers. The insets show the Laue diffraction patterns. The theoretical pattern for Al_12_CaO_19_ (CA6) is given for comparison.

The patterns for the samples after HPHT treatment between graphene layers are shown in Figure 4. For the C12A7-600 sample (Experiment 1), the patterns correspond well to the theoretical one for the CA6 (CaO∙6Al_2_O_3_) phase with lattice parameters of *a* = 5.5587 and *c* =21.8929 (PDF card #01-084-1613; ICDD card #202616). A number of diffraction reflections in the low-angle region can be attributed to the carbon-containing phase of the Al_4_O_20_C_61_._8_ (COD 96-410-3389), but unidentified lines remain. These data differ significantly from the results of similar experiments for mayenite described by Murata et al. [49], where the destruction of its structure under HPHT conditions was accompanied only by the formation of calcium aluminates of C2A and C4A3 compositions. It can be supposed that the presence of contact with graphene under the conditions of HPHT experiments not only leads to the formation of an aluminum-enriched CA6 phase but is also accompanied by the appearance of a whole spectrum of carbide-like compounds, the identification of which is difficult.

As it follows from the pattern for the C12A7@C-1400 sample after HPHT treatment between graphite layers (Experiment 2) shown in Figure 4, mono- and polycrystalline components are present in the sample. The presence of local diffraction reflections and diffraction arcs indicates this. Identification of the phase composition of the resulting sample was not possible due to the lack of information about such structures in the databases. As is assumed, a number of previously unidentified carbide-like compounds may also be included. It is important to note that as a result of the HPHT treatment of the C12A7@C-1400 sample, no known calcium aluminate phases appear at all. This situation differs significantly from that described above for the C12A7-600 sample, despite the fact that in both cases, mayenite can react with carbon only.

### 3.3. Characterization of the C12A7-600 and C12A7@C-1400 Samples after the HPHT Experiment between MgO Layers

It has previously been noted that graphite and MgO chosen as buffer layers for the HPHT experiments do not react under normal conditions (vacuum or atmospheric pressure) with mayenite up to 1500 °C. For this reason, they can be considered inert substrates to create films of the electride on their surface, the formation of which requires high temperatures. According to the results presented above, already at a pressure of 4 GPa and a temperature of 1450 °C, graphite is not an inert material for mayenite and is able to initiate a number of its solid-phase transformations.

**Figure 5:**
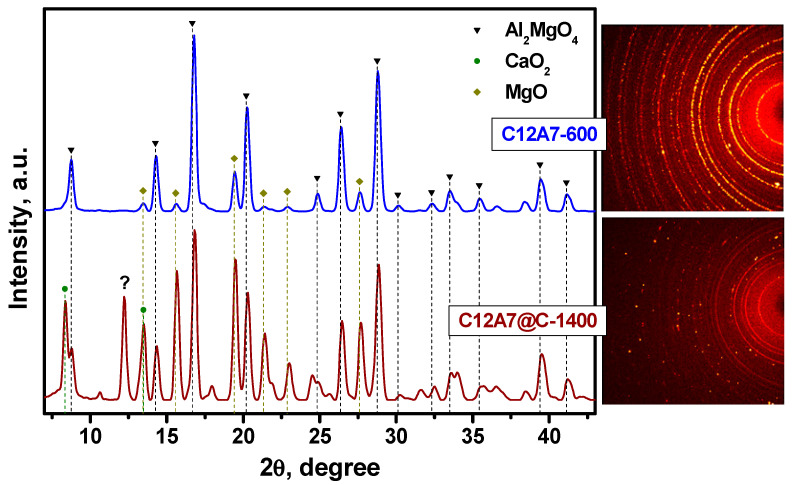
X-ray patterns of the C12A7-600 and C12A7@C-1400 samples after the HPHT experiments with the MgO buffer layers. The insets show the Laue diffraction patterns. An unidentified peak is labeled by the question mark (“?”).

It was assumed that the replacement of graphite with MgO under conditions similar to HPHT experiments will also be accompanied by solid phase reactions involving mayenite. Figure 5 shows the patterns for the samples after the HPHT treatment between MgO layers. In the case of pure mayenite (Experiment 2), the main reflections on the pattern correspond to the spinel Al_2_MgO_4_ phase (*a* = 8.088 Å, space group *Fd-3m*). The remaining reflections are referred to as the MgO phase (buffer layer material). No other phases involving Ca were detected, probably they are amorphous.

The C12A7@C-1400 sample behaved somewhat differently with treatment under the HPHT conditions between MgO layers. The corresponding pattern is represented by the reflections assigned to Al_2_MgO_4_, MgO, and CaO_2_ (portlandite; *a* = 3.568; *c* = 4.863; space group No. 164). Among the unidentified reflections, there is a rather intense one, which is indicated by the question mark. The presented data indicate that the presence of a carbon coating under the conditions of HPHT experiments, although it does not interfere with the interaction of the oxide core of the C12A7 with the material of the MgO buffer layers, significantly affects the solid-phase products resulting from the reaction.

## 4. Conclusions

The literature to date has shown that mayenite (C12A7) does not react substantially under normal conditions (vacuum or inert gas atmosphere) with graphite and MgO up to 1500 °C. For this reason, such materials can act as inert substrates to create films of the electride on their surface, the formation of which requires high temperatures. The present paper presents the results of the study of solid-phase transformations under HPHT conditions (P = 4 GPa, T = 1450 °C) of pure mayenite and core-shell structure C12A7@C with graphite and MgO in order to assess the effect of the deposited carbon shell on the course of such reactions. It has been found that the presence of contact of pure C12A7 with graphite under HPHT conditions leads to the formation of aluminum-enriched calcium aluminates (CA6), while core-shell structure C12A7@C under similar conditions form a whole spectrum of hardly identifiable phases of calcium aluminates and carbide-like phases. Note that the CA6 phase is absent in such samples. It has been shown that the main result of the interaction of pure mayenite C12A7 and core-shell structure C12A7@C with MgO under HPHT conditions is the appearance of the Al_2_MgO_4_ spinel phase. This means that the carbon shell in the C12A7@C sample is not able to prevent the reaction of the mayenite oxide core with MgO outside this structure under such conditions. However, the other solid phase products of the mayenite reaction with MgO accompanying this spinel are significantly different for pure C12A7 and core-shell structure C12A7@C. Thus, it can be concluded that the conditions used in the HPHT experiments in all cases lead to the complete destruction of the mayenite structure and the formation of new phases, the composition of which differs significantly when using mayenite C12A7 and core-shell structures C12A7@C. An important result of the study is the discovery of the possibility of forming carbide phases during the interaction of mayenite with graphite under the used HPHT conditions.

## Data Availability

Data is contained within the article.
